# Molecular insights into the regulation of flavonoid biosynthesis in fruits

**DOI:** 10.1093/hr/uhaf306

**Published:** 2025-11-02

**Authors:** Lili Chen, Yuan Cheng, Gaojie Hong

**Affiliations:** State Key Laboratory for Quality and Safety of Agro-Products, Key Laboratory of Biotechnology in Plant Protection of MARA, Key Laboratory of Green Plant Protection of Zhejiang Province, Institute of Virology and Biotechnology, Zhejiang Academy of Agricultural Sciences, Hangzhou 310021, China; State Key Laboratory for Managing Biotic and Chemical Threats to the Quality and Safety of Agro-Products, Vegetable Research Institute, Zhejiang Academy of Agricultural Sciences, Hangzhou 310021, China; State Key Laboratory for Quality and Safety of Agro-Products, Key Laboratory of Biotechnology in Plant Protection of MARA, Key Laboratory of Green Plant Protection of Zhejiang Province, Institute of Virology and Biotechnology, Zhejiang Academy of Agricultural Sciences, Hangzhou 310021, China

## Abstract

Flavonoids are important secondary metabolites that regulate plant growth and development and confer resistance against biotic and abiotic stress. As natural polyphenol substances, flavonoids determine the quality traits of commercial fruits, such as color, flavor, and nutrition. In the past few decades, research on the regulation of flavonoid biosynthesis in plants has made significant progress. However, a deep understanding of this aspect in flavonoid-rich horticultural crops is lacking. This review aims to systematically summarize the current knowledge in the regulation of flavonoid biosynthesis in fruits, including the transcriptional, post-transcriptional, epigenetic, and post-translational regulation mechanisms as well as the composite regulation cascades. Our analysis shows that direct transcriptional regulation involves the actions of different transcription factor families, such as MYB, WRKY, bZIP, AP2/ERF, and MADS, by directly targeting the key synthase genes in flavonoid biosynthetic pathway. Indirect regulation involves specific transcription factors and microRNAs that target the downstream regulators, as well as the regulation modules triggered for degradation of activators or repressors in response to environmental signals or plant hormones. In addition, epigenetic regulation, associated with methylation level in the gene promoter regions or the insertion or deletion of specific sequences therein, plays an important role in controlling anthocyanin accumulation. Based on the diverse regulation mechanisms of the flavonoid biosynthetic pathway, more molecular design targets can be applied in the future, facilitating the production of more stress-tolerant and quality-elevated crop varieties.

## Introduction

**Figure 1:**
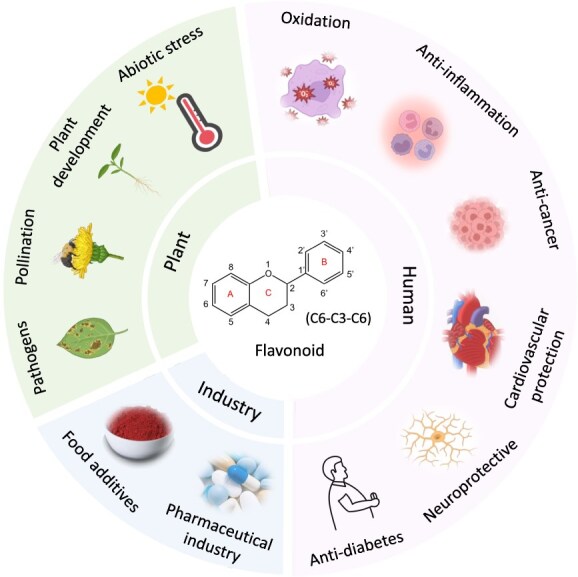
Function and application of flavonoids.

Flavonoids are important bioactive compounds in food plants that are characterized by the C_6_-C_3_-C_6_ basic structure (Fig. 1). They specifically determine the quality of fruits by their diverse effects on color, aroma, taste, and antioxidant properties [1]. Flavonoids regulate plant development and defense, and they affect a series of biological processes, such as auxin transport, root development, pollination ultraviolet (UV) protection, free-radical scavenge, and the resistance against herbivory and pathogen attack [2–5] (Fig. 1). Moreover, flavonoids have strong antioxidant activity, and they exert protective effects against a series of human chronic diseases such as diabetes, cardiovascular disorders, cancer, and neurodegenerative conditions [6–8] (Fig. 1).

**Figure 2:**
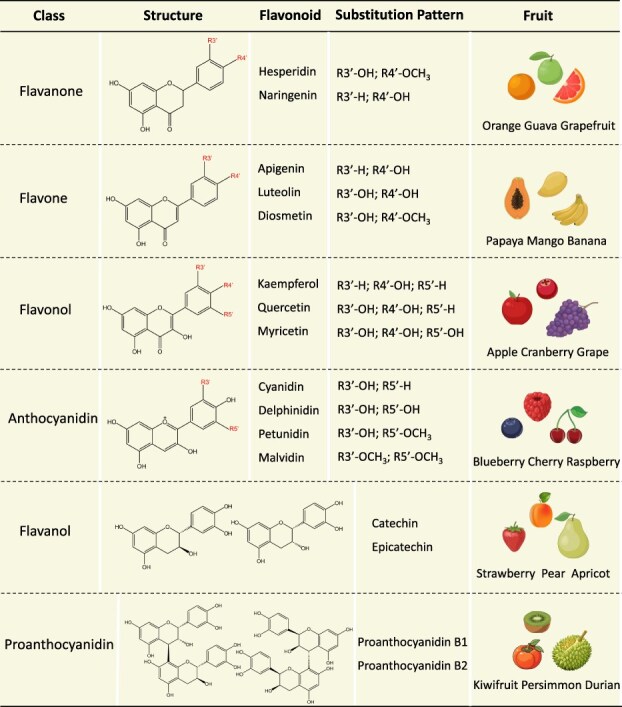
Aglycone class and chemical structures of flavonoids in fruits.

Six classes of flavonoid compounds are present in commercial fruits, including flavonols, anthocyanins, flavanones, flavones, flavanols, and proanthocyanidins (PAs) (Fig. 2). Fruits are important resources for dietary intake of flavonoids, and those rich in various flavonoid aglycones and their glycosylated forms have been widely studied and reported in recent years [9–21] (Table 1). In many fruits, flavonols and PAs accumulate early during fruit development. In contrast, anthocyanin accumulation often indicates ripening [22]. Based on their economic importance and health-promoting effects, the extensively studied fruits include apple (*Malus domestica*), pear (*Pyrus communis*), blueberry (*Vaccinium corymbosum*), citrus (*Citrus sinensis*), strawberry (*Fragaria ananassa*), grape (*Vitis vinifera*), and kiwifruit (*Actinidia chinensis*) [23]. In the past few decades, considerable research advances have been made in demonstrating the biosynthesis and regulation of flavonoids in fruits [22, 24]. The composition of flavonoids and qualitative or quantitative changes in these compounds are mainly controlled by the interactions between the genetic background of fruits and various external and internal factors, such as light, temperature, fertilizers, physical wounds, biotic stress, and hormones [25]. The key players in the underlying mechanisms involve transcription factors (TFs), protein complexes, microRNAs (miRNAs), and regulatory cascades [22]. However, the systematic review demonstrating these regulatory mechanisms is lacking.

**Table 1:** The content of flavonoid in the edible portion of fruits.

**Species**	**Class**	**Flavonoids**	**Content (μg/g FW)**	**Reference**
Apple, peel (*Malus domestica*)	Flavonol	Quercetin 3-galactoside	57.8–100.9	[9]
		Quercetin 3-glucoside	11.8–89.2	
		Quercetin 3-xyloside	20.3–44.9	
		Quercetin 3-arabinoside	43.5–103.3	
		Quercetin 3-rhamnoside	32.3–67.4	
		Quercetin	4980–19 670	
		Kaempferol	3690–14 250	
	Anthocyanin	Cyanidin 3-galactoside	0–208.2	
	Flavanol	Catechin	4590–18 610	
		Epicatechin	2140–7590	
Grape (*Vitis vinifera*)	Flavonol	Myricetin 3-galactoside/glucoside	6.5–101.9	[10]
		Quercetin 3-galactoside/glucoside	0–75.5	
		Astragalin	2.0–94.0	
		Hyperoside	63.4–287.7	
		Isorhamnetin 3-O-glucosid	10.2–317.2	
		Isorhamnetin-3-Oneohespeidoside	19.9–218.3	
		Miquelianin	328.4–1198.8	
		Myricetin	5.9–138.0	
		Quercimeritrin	10.7–18.0	
		Quercitrin	0.3–12.4	
		Rutin	60.1–298.3	
	Anthocyanin	Malvidin	3968.7–6910.6	
		Petunidin	470.8–2183.7	
		Deiphinidin	303.5–1739.4	
		Peonidin	146.5–609.8	
		Cyanidin	31.3–220.9	
		Pelargonidin	1.2–16.3	
	Flavanol	Catechin	6.0–22.9	
		Epicatechin	11.1–44.7	
		Epigallocatechin	11.0–37.6	
		Gallocatechin	10.0–42.3	
	Flavanone	Naringenin-7-glucoside	1.5–4.4	
	Flavone	Diosmin	42.0–218.5	
		Limocitrin	15.5–28.6	
		Narcissin	19.5–186.6	
Mandarin orange (*Citrus suhuiensis*)	Flavanone	Naringenin-7-*O*-rutinoside	12 863–13 395^a^	[11]
		Hesperetin-7-*O*-rutinoside	2742–2769^a^	
		Isosakuranetin-7-*O*-rutinoside	108–387^a^	
	Flavone	Apigenin-6,8-di-*C*-glucoside	95–102^a^	
		Diosmetin-6,8-di-*C*-glucoside	470–474^a^	
		Chysoeriol-6,8-di-*C*-glucoside	245–259^a^	
Pear (*Pyrus communis*)	Flavonol	Quercetin-3-rutinoside	0.2–0.5	[12]
		Quercetin-3-galactoside	1.0–1.1	
		Quercetin-3-glucoside	0.6–3.5	
		Isorhamnetin-3-glucoside	0.9–9.1	
		Quercetin	3280–14 570	
		Kaempferol	1280–8590	
	Flavanol	Catechin	4590–17 450	
		Epicatechin	1580–6980	
Strawberry (*Fragariaananassa*)	Flavonol	Quercetin-3-*O*-rutinoside	4.1–96.7	[13]
	Flavanol	Catechin	26.5–85.3	
		Epicatechin	12.8–93.2	

In this review, we provide molecular insights into the regulation of flavonoid biosynthesis in fruits by addressing transcriptional, post-transcriptional, epigenetic, and post-translational regulation mechanisms as well as complex regulation modules. TFs, miRNAs, and transcriptional regulatory cascades will be summarized and discussed. Direct transcriptional regulation of fruit flavonoids involves TFs and protein complexes, such as MYB, WRKY, bZIP, AP2/ERF, MADS, and MBW (MYB-bHLH-WDR) complex, which directly target the structural genes within the flavonoid biosynthetic pathway. In addition, factors that regulate the MBW complex and the mechanism of the feedback regulation of the MBW complex will be discussed. Epigenetic regulation of anthocyanin biosynthesis extensively exists in fruits, which is achieved by the alterations of the methylation level and the insertion of transposons or the deletion of specific sequences in the promoter region of the key regulatory genes. Diverse regulatory pathways will be described and summarized, which involve the miRNAs targeting the key TFs, the protein–protein interactions between regulators, and the degradation of activators or repressors during the signaling response induced by light or phytohormones. To sum up, this review provides comprehensive insights into the production and regulation of flavonoids in fruits. The theoretical basis addressed will pave the way for genetic engineering of horticultural crops and the *de novo* production of flavonoids *in vitro*.

## Flavonoid biosynthetic pathway

Flavonoid accumulation in plants requires the action of two distant biosynthetic pathways, namely the shikimate pathway and the acetate pathway. The shikimate pathway provides the *p*-Coumaroyl-CoA substrate that forms ring B and the acetate pathway generates malonyl-CoA that forms ring A of the flavonoid. These components merge through the linking ring C to form the C_6_-C_3_-C_6_ backbone of flavonoid, and it is achieved by the catalytic activity of chalcone synthase (CHS) and chalcone isomerase (CHI) [26].

**Figure 3:**
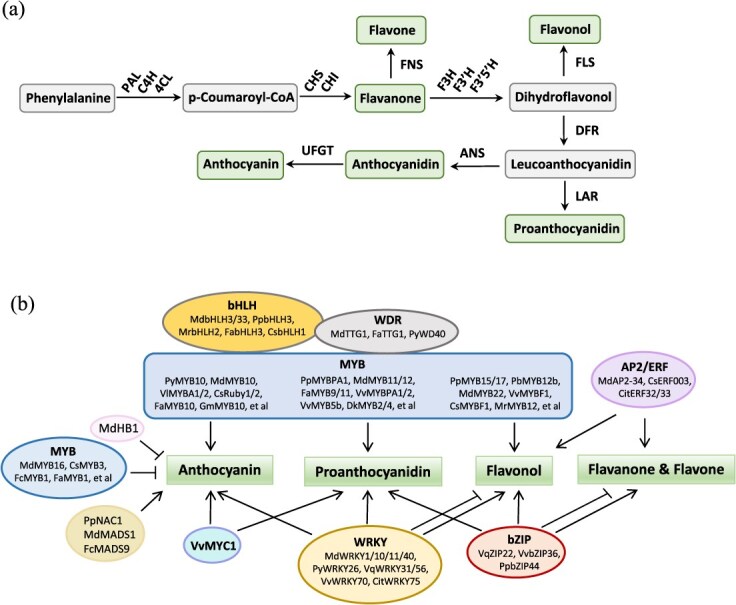
Flavonoid biosynthetic pathway and the direct transcriptional regulation of flavonoid biosynthesis in fruits. (a) Flavonoid biosynthetic pathway. PAL, phenylalanine ammonia lyase; C4H, cinnamate 4-hydroxylase; 4CL, 4-coumaroyl CoA ligase; CHS, chalcone synthase; CHI, chalcone isomerase; FNS, flavone synthase; F3H, flavanone 3-hydroxylase; F3′H, flavonoid 3′-hydroxylase; F3′5′H, flavonoid 3′5′-hydroxylase; FLS, flavonol synthase; UFGT, uridine diphosphate (UDP)-glucose flavonoid-3-O-glycosyltransferase; DFR, dihydroflavonol 4-reductase; ANS, anthocyanidin synthase; LAR, leucoanthocyanidin reductase. (b) TFs that directly regulate flavonoid biosynthesis in fruits. Arrows and blunt ends represent promotion and inhibition effects, respectively.

Phenylalanine is the precursor amino acid in the flavonoid biosynthetic pathway (Fig. 3a). Phenylalanine ammonia-lyase (PAL) mediates the deamination of phenylalanine to generate cinnamic acid. Subsequently, cinnamic acid hydroxylase (C4H) hydroxylates cinnamic acid to produce *p*-coumaric acid [27]. Next, *p*-coumaric acid is catalyzed by coumarin CoA ligase (4CL) in a condensation reaction to synthesize *p*-coumaroyl CoA. Malonyl CoA, which comes from the acetate pathway, is involved in the following reaction. The step-by-step condensation of malonyl CoA with *p*-coumaroyl CoA is then catalyzed by CHS to produce naringenin chalcone [28]. Ultimately, CHI converts naringenin chalcone into naringenin or flavanone [26]. Naringenin is the common precursor for synthesizing different flavonoids. Various structural modifications of naringenin generate different classes of flavonoids such as flavones, isoflavones, dihydroflavonols, flavonols, and anthocyanidins [26]. Isoflavone synthase (IFS) and flavone synthase (FNS) catalyze the formation of isoflavone and flavones, respectively, from naringenin (flavanone). Flavanone-3-hydroxylase (F3H) converts naringenin into dihydroflavonol. The subsequent biosynthesis of leucoanthocyanidins from dihydroflavonol relies on the dihydroflavonol 4-reductase (DFR) [29]. Leucoanthocyanidin dioxygenase (LDOX) and anthocyanidin synthase (ANS) can catalyze leucoanthocyanidins to produce anthocyanidins. The subtract anthocyanidins then react with uridine diphosphate (UDP)-glucose flavonoid-3-*O*-glycosyltransferase (UFGT) to produce anthocyanin. Alternatively, the leucoanthocyanidin reductase (LAR) catalyzes the transition of leucoanthocyanidin to flavanols, and the flavanol units can be polymerized into proanthocyanins. Catalyzed by flavonol synthase (FLS), dihydroflavonol can be converted into flavonols, such as kaempferol, myricetin, and quercetin.

**Table 1:** Continued.

**Species**	**Class**	**Flavonoids**	**Content (μg/g FW)**	**Reference**
	Anthocyanin	Cyanidin-3-glucoside	35.3–88.4	
		Pelargonidin 3-glucoside	107.9–420.2	
		Pelargonidin 3-rutinoside	17.1–81.9	
	Procyanidin	Procyanidin B2	0–89.5	
Kiwifruit (*Actinidia chinensis* spp.)	Flavonol	Kaempferol	80.9^a^	[14]
		Quercetin	18.6^a^	
		Quercetin-3-*O*-galactoside	205.1–470.9^a^	
	Flavanone	Naringenin	24.7^a^	
	Flavone	Apigenin 7-glucoside	3.4^a^	
	Dihydrochalcone	Phloridzin	2-5^a^	
	Anthocyanin	Total anthocyanin	13.7–40.5	
	Procyanidin	Procyanidin B1	64.6–446.8^a^	
		Procyanidin B2	17.8–182.1^a^	
Sweet cherry (*Prunus avium*)	Flavone	Luteolin	11.1–25.4	[15]
	Flavonol	Quercetin-3-*O*-glucoside	7.0–13.6	
		Quercetin-3-*O*-rutinoside	8.9–14.2	
		Kaempferol	14.3–19.8	
	Anthocyanin	Cyanidin-3-*O*-glucoside	0–38.4	
		Cyanidin-3-*O*-rutinoside	0–850.1	
Chinese bayberry (*Myrica rubra* Sieb. et Zucc)	Flavonol	Myricetin-3-*O*-rhamnoside	10.7–50.7	[16]
		Quercetin-3-*O*-galactoside	0.1–74.5	
		Quercetin-3-*O*-glucoside	0.1–9.1	
		Quercetin-3-*O*-rhamnoside	3.3–51.7	
	Anthocyanin	Cyanidin-3-*O*-glucoside	9.3–837.3	
Watermelon (*Citrullus vulgaris*)	Flavonol	Quercetin	10	[17]
		Kaempferol	10	
		Morin	30	
Papaya (*Carica papaya*)	Flavonol	Myricetin	11 300–17 100^a^	[18]
		Quercetin	2380-2510^a^	
		Kaempferol	5810-17 990^a^	
		Quercetin-3-*O*-rutinoside	4320–8190^a^	
	Flavone	Apigenin	3740-9560^a^	
		Luteolin	590-790^a^	
Mango (*Mangifera indica*)	Flavonol	Myricetin	960–21 080^a^	[18]
		Quercetin	1030-1690^a^	
		Kaempferol	2300-10 140^a^	
		Quercetin-3-*O*-rutinoside	231 790–1 037 580^a^	
	Flavone	Apigenin	1620-2070^a^	
		Luteolin	100-600^a^	
Pineapple (*Ananas comosus*)	Flavonol	Myricetin	4.8–6.5	[19]
		Quercetin	3.9–4.8	
		Kaempferol	20.5–25.1	
	Flavone	Apigenin	4.4–16.7	
		Luteolin	0.9–4.4	
	Flavanone	Hesperetin	3.5–15.4	
Blueberry (*Vaccinium corymbosum*)	Flavonol	Myricetin 3-galactoside/glucoside	22.5–40.2	[20]
		Myricetin 3-rhamnoside	3.4–14.3	
		Quercetin 3-galactoside/xyloside	56.0–134.9	
		Quercetin 3-glucoside/rutinoside	0–105.7	
		Quercetin 3- acetylrhamnoside	14.3–91.5	
	Anthocyanin	Cyanidin 3-galactoside/arabinoside	61.7–1111.9	
		Delphinidin 3-galactoside/arabinoside	351.6–2178.8	
		Petunidin 3-glucoside/arabinoside	121.2–423.3	

**Table 1:** Continued.

**Species**	**Class**	**Flavonoids**	**Content (μg/g FW)**	**Reference**
		Petunidin 3-galactoside/acetylglucoside	129.1–1248.1	
		Peonidin 3-galactoside/arabinoside	20.2–370.6	
		Malvidin 3-galactoside/acetylglucoside	195.9–1802.6	
		Malvidin 3-arabinoside	105.2–718.6	
Cranberry (*Vaccinium oxycoccos*)	Flavonol	Myricetin derivatives	4960–9260^a^	[21]
		Quercetin derivatives	1070–2250^a^	
		Methoxyquercetin derivatives	333.1–430.4^a^	
	Flavanol	Catechin	27.9-75.3^a^	
		Epicatechin	274.6-608^a^	
	Anthocyanin	Delfinidyn derivatives	312.7–438.7^a^	
		Cyanidin derivatives	4420–9670^a^	
		Peonidin derivatives	1920–6660^a^	
		Malvidin derivatives	298.5–588.5^a^	
	Procyanidin	Polymeric PAs	6510^a^-11 090	
Banana (*Musa sapientum*)	Flavonol	Myricetin	880–900^a^	[18]
		Quercetin	510-14 540^a^	
		Kaempferol	790-860^a^	
		Quercetin-3-*O*-rutinoside	7870–9700^a^	
	Flavone	Apigenin	830-840^a^	
		Luteolin	700-14 960^a^	
Durian (*Durio zibethinus*)	Flavonol	Myricetin	8.4–30.4	[19]
		Kaempferol	1.0–10.6	
	Flavone	Apigenin	4.7–11.0	
		Luteolin	2.1–5.5	
	Flavanone	Hesperetin	2.3–13.2	
Guava (*Psidium guajava*)	Flavonol	Myricetin	22.1–25.3	[19]
		Quercetin	33.2–36.0	
		Kaempferol	0.5–7.7	
	Flavone	Apigenin	4.0–5.1	
		Luteolin	10.4–15.4	
	Flavanone	Hesperetin	8.0–29.1	

FW, fresh weight.

a Dry weight basis.

## Transcriptional regulation of flavonoid biosynthesis

### MYB transcription factors and MBW complex

TFs involved in regulating the flavonoid biosynthesis pathway in fruits include MYB, bHLH, WD40, MYC, bZIP, AP2/ERF, WRKY, and MADS-box proteins [30–100] (Fig. 3b, Table 2). Among those TFs, MYB proteins are extensively studied. Some MYBs are specific for regulating anthocyanin in fruits. PyMYB10 induced anthocyanin biosynthesis in Asian pear (*Pyrus pyrifolia*) [30]. MdMYB10 can induce anthocyanin accumulation in apples (*M. domestica*). The red apple variety ‘Red Field’ had high expression of *MdMYB10* in the cortex, thus differentiating it from the other white-fleshed cultivars. The induction of *MdMYB10* expression was concurrent with color formation in either the flesh tissue or the skin of specific apple varieties [31]. Besides, MdMYB10 can bind and activate its own promoter via the upstream repeat units for protein production [101]. There are three MYB alleles (MdMYB10, MdMYB1, MdMYBA) that control the red pigmentation of apples [32–34, 102]. MdMYB1, MdMYBA, and MdMYB3 are involved in anthocyanin biosynthesis in apple skin. While MdMYB10 controls both the skin and the flesh pigmentation [32, 33, 35, 102]. In grapevine, light induced an array of MYB TF expressions for positively regulating the general flavonoid pathway and for specifically regulating anthocyanin (*VlMYBA1*, *VlMYBA2*), flavonol (*VvMYBF1*, *VvMYB12*), and PAs (*VvMYBPA1*, *VvMYBPA2*) [2, 36, 103, 104]. *VlMYBA1* and *VlMYBA2* genes played a critical role in regulating anthocyanin biosynthesis in the grape (*Vitis labruscana*) via control of *UFGT* gene expression [37]. MYB TFs, CsRuby1 and CsRuby2, can activate anthocyanin accumulation in a range of *Citrus* species [38, 39]. FaMYB10 regulated flavonoid metabolism in ripened strawberry fruits (*F. ananassa*). *FaMYB10* expression in the fruit receptacles was regulated by the hormone auxin and abscisic acid (ABA). Transient silence of *FaMYB10* expression significantly inhibited anthocyanin production [40]. In nectarines (*Prunus persica*), PpMYB10 positively regulated anthocyanin via the transactivation of *UFGT* and *DFR* [41]. GmMYB10 and MrMYB1 regulate anthocyanin biosynthesis in mangosteen and Chinese bayberries, respectively [42, 43].

PA-specific MYBs. VvMYBPA1 and VvMYBPA2 are specific for the regulation of PA biosynthesis in grapes, and they activated the promoters of *VvLAR1* and *VvANR* [2, 36]. PpMYBPA1 regulated PA synthesis by controlling *DFR* and *LAR* in nectarine [41]. Persimmon (*Diospyros kaki*) is capable of accumulating abundant PAs in the flesh. DkMYB4 regulated PA biosynthesis in persimmon by directly acting on the promoters of PA pathway genes [44]. DkMYB2 was another regulator of PA synthesis, and it drove transcriptional activation of *DkANR* and *DkLAR* [45].

Flavonol-specific MYBs. PbMYB12b promoted the production of quercetin glycosides and isorhamnetin glycosides by activating the expression of *PbCHSb* and *PbFLS* [46]. MdMYB22 activated flavonol pathways in apples [47]. VvMYBF1 specifically activated the transcription of flavonol synthase1 (*VvFLS1*) and promoted flavonol synthesis in the grapevine (*V. vinifera*). *VvMYBF1* expression was light-inducible, and it correlated with *VvFLS1* expression and flavonol accumulation [48]. In sweet orange, CsMYBF1 controlled both flavonol and hydroxycinnamic acid biosynthesis [49]. In peach fruit, two R2R3-MYB TFs, PpMYB15 and PpMYBF1, specifically regulated flavonol biosynthesis [50].

MYBs involved in regulating several kinds of flavonoids. PbMYB10b regulated the biosynthesis of anthocyanin and PA by inducing *PbDFR* expression [51]. PbMYB9 not only specifically activated the PA pathway by acting on *PbANR*, but also stimulated anthocyanins and flavonol production by regulating *PbUFGT1* [51]. PpMYB17 regulated flavonol biosynthesis and other flavonoid accumulation [52]. MdMYB9 positively regulated PA synthesis, and it activated the anthocyanidin reductase (*ANR*) promoter [53]. Induced by methyl jasmonate (MeJA), MdMYB9 and MdMYB11 promoted anthocyanin and PA accumulation in apple calli [54]. Overexpression of *VvMYB5a* strongly induced anthocyanin and flavonol compounds in tobacco [55]. Like VvMYB5a, VvMYB5b promoted anthocyanins and PA production in the flowers of transgenic tobacco [56].

Central to the regulation of flavonoid biosynthesis in many species is a transcriptional complex of MBW proteins [57, 105, 106]. MBW complex is essential for activating the late steps of flavonoid biosynthesis, and it has been characterized in many fruits, such as apples [31], grapes [58], and strawberries [59]. MdMYB12 could interact with bHLH3 and bHLH33 to enhance PA synthesis [45]. MdMYB9 and MdMYB11 proteins bound to the promoters of *MdANS*, *MdANR*, and *MdLAR*, and they interacted with MdbHLH3 [54]. MdMYB10 promoted anthocyanin synthesis, and its homologs have been isolated from other major rosaceous species, such as loquat (EjMYB10), apricot (ParMYB10), Japanese plum (PsMYB10), sweet cherry (PavMYB10), and red raspberry (RiMYB10) [34]. Rosaceous MYB10s could induce anthocyanin biosynthesis in transient assays, but they required the coexpression of bHLH proteins [34]. In strawberries, FaMYB9/11-FabHLH3-FaTTG1 was proposed to regulate PA biosynthesis [59]. The PyMYB10-PybHLH-PyWD40 complex regulated anthocyanin accumulation in the peel of Yunnan red pear [107]. By interacting with PpbHLH3, both PpMYB10 and PpMYB114 could form the MBW complex to regulate anthocyanin biosynthesis in pear fruit [60, 61, 108]. In Chinese bayberry (*Morella rubra*), MrMYB12 upregulated quercetin biosynthesis by activating the promoter of *MrFLS2*. The synergistic actions with the other two TFs, MrMYB5L and MrbHLH2, induced a higher accumulation of myricetin derivatives [62].

MYB repressors of flavonoid biosynthesis. The repression activities of MYBs can be achieved by competitive and nonproductive binding to promoters of structural genes or suppressing the activity of bHLHs or MBW via interacting with them [109]. Some repressors have repression domains like the C2 motif and EAR (ethylene-responsive element-binding factor-associated amphiphilic repression) motif in the C-terminal region [110]. MdMYB6, MdMYB16, and MdMYB15L negatively regulated anthocyanin biosynthesis in apples [63–66]. FaMYB1 repressed flavonol (quercetin) and anthocyanin pathways in transgenic tobacco [67]. FcMYB1 is an ortholog of FaMYB1 isolated from the white Chilean strawberry. It repressed the anthocyanin pathway and regulated the branching point of the anthocyanin/PA biosynthesis [68]. Other MYBs that negatively regulate anthocyanin or flavonoid biosynthesis include VvMYB4-like and VvMYBC2-L1/L2/L3 in grapes, and PpMYB17-20 in peach flowers [57, 69–71].

**Table 2:** Transcriptional regulation of flavonoid biosynthesis in fruits.

**Species**	**Transcription factor**	**Regulatory effect**	**Reference**
Pear (*P. pyrifolia*)	PyMYB10, PpMYB10/114, PyWRKY26, PybHLH3	Anthocyanin (+)	[30, 60, 61, 90]
	PyMYB107	Anthocyanin (−)	[93]
	PbMYB12b	Flavonol (+)	[46]
	PbMYB10b	Anthocyanin (+), PA(+)	[51]
	PbMYB9	Anthocyanin (+), PA(+), flavonol (+)	[51]
	PpMYB17	Anthocyanin (+), flavonols (+), flavanones (+), flavones (+), isoflavones (+)	[52]
	PpbZIP44	Flavonols (+), flavones (+), Isoflavones (+), dihydroflavones (+), chalcones (+)	[78]
Apple (*M. domestica*)	MdMYB1/3/10, MdMYBA, MdWRKY10/11/40, MdWRKY1, MdERF109, MdMADS1	Anthocyanin (+)	[31–33, 35, 72, 82, 84, 94, 96]
	MdCOP1, MdHB1	Anthocyanin (−)	[88, 95]
	MdMYB6/16/15 L	Anthocyanin (−)	[63–66]
	MdAP2–34	Flavonol (+)	[81]
	MdMYB9/11	Anthocyanin (+), PA (+)	[53, 54]
	MdHY5	Anthocyanin (+), flavonol (+)	[97]
	MdMYB12/22	Flavonol (+), PA (+)	[47]
Grape (*V. vinifera* )	VlMYBA1/A2	Anthocyanin (+)	[37]
	VvMYB4-like, VvMYBC2-L1/L2/L3, VvBBX44	Anthocyanin (−)	[57, 69, 70, 92]
	VvMYBPA1/2, VqWRKY56, VqbZIPC22	PA (+)	[2, 36, 74]
	VvMYBF1	Flavonol (+)	[48]
	VvWRKY70	Flavonol (−)	[73]
	VvMYB5b, VvMYC1	Anthocyanin (+), PA (+)	[56, 58]
	VvMYB5a	Anthocyanin (+), flavonol (+)	[55, 56]
	VvibZIPC22	Anthocyanin (+), PA (+), flavonol (+)	[98]
	VvbZIP36	Anthocyanin (−), flavones (−), flavonol (+)	[77]
	VqWRKY31	Flavanones (+), flavonol (+)	[75]
Citrus (*C. sinensis* )	CsRuby1/2, CitWRKY75	Anthocyanin (+)	[38, 39, 85]
	CsMYB3	Anthocyanin (−)	[91]
	CsMYBF1	Flavonol (+)	[49]
	CsERF003	Flavanones (+), flavonols (+)	[80]
	CitERF32/33, CitRAV1	Flavanones (+), flavones (+)	[79]
Strawberry (*F. ananassa*)	FaMYB10	Anthocyanin (+)	[40]
	FcMYB1	Anthocyanin (−)	[68]
	FaMYB9/11, FabHLH3, FaTTG1	PA (+)	[59]
	FaMYB1	Anthocyanin (−), flavonol (−)	[67]
Peach (*P. persica*)	PpMYB10, PpBL, PpNAC1	Anthocyanin (+)	[71, 87]
	PpMYB17–20	Flavonoid (−)	[41]
	PpMYBA1	PA (+)	[41]
	PpMYB15, PpMYBF1	Flavonol (+)	[50]
Kiwifruit (*Actinidia* sp.)	AcMYB10/110	Anthocyanin (+)	[99]
	AcMADS68	Anthocyanin (+)	[89]
	AcWRKY44, AcMYBC1	Anthocyanin (+), PA (+)	[76]
Chinese bayberry (*M. rubra*)	MrMYB1	Anthocyanin (+)	[42]
	MrMYB5/5 L/12	Flavonol (+)	[62]

**Table 2:** Continued.

**Species**	**Transcription factor**	**Regulatory effect**	**Reference**
Persimmon (*D. kaki*)	DkMYB2/4	PA (+)	[44, 45]
Mangosteen (*Garcinia mangostana*)	GmMYB10	Anthocyanin (+)	[43]
Loquat (*Eriobotrya japonica*), Apricot (*Prunus armeniaca*), Plum (*Prunus salicina*), Cherry (*P. avium*), Red Raspberry (*Rubus idaeus*)	EjMYB10, ParMYB10, PsMYB10, PavMYB10, RiMYB10	Anthocyanin (+)	[34]
Bilberry (*Vaccinium myrtillus*)	VmTDR4	Anthocyanin (+)	[86]
Fig (*F. carica*)	FcMADS9	Anthocyanin (+)	[83]
Litchi (*Litchi chinensis*)	LcMYB1	Anthocyanin (+)	[100]

The sign of plus (+) and minus (−) represents activation and inhibition effects, respectively.

### WRKY, bZIP, AP2/ERF, MADS transcription factors

The functions of WRKY and bZIP TFs in flavonoid regulation have been demonstrated in recent years. *MdWRKY11* overexpression upregulated anthocyanin and flavonoid accumulation in apple calli [72]. VvWRKY70 repressed flavonol biosynthesis in grape berries. Environmental signals such as light and high temperature could downregulate *VvWRKY70* expression [73]. Overexpression of either *VqWRKY31* or *VqWRKY56* in grapevine enhanced powdery mildew (PM) resistance and increased the level of salicylic acid and reactive oxygen species (ROS) [74, 75]. VqWRKY31 promoted the biosynthesis of flavanones and flavonols [75], and overexpression of *VqWRKY56* increased PA content [74]. In kiwifruit, both WRKY44 and MYBC1 could increase PA levels in the calli [76]. VqbZIPC22 interacted with VqWRKY56 *in planta* and synergistically promoted PA-mediated PM resistance in grape leaves [74]. Knocking out of *VvbZIP36* with Clustered Regularly Interspaced Short Palindromic Repeats (CRISPR) and CRISPR-associated protein 9 (Cas9) promoted anthocyanin accumulation in leaves and inhibited flavonol synthesis [77]. In pear fruit, PpbZIP44 positively regulated primary and secondary metabolism, and it directed carbon flux toward the accumulation of flavonoid and phenylalanine metabolites [78].

By activating chalcone isomerase expression, three AP2/ERF TFs enhanced flavanone and flavone accumulation in citrus [79]. In navel orange, CsERF003 could specifically direct the carbon flux for flavonoid synthesis [80]. In response to visible light, MdAP2-34 stimulated flavonol accumulation in apple flesh via activating *MdF3′H* [81]. MdERF109 promoted anthocyanin biosynthesis by directly binding to the promoters of *MdCHS*, *MdUFGT*, and *MdbHLH3* [82]. In figs, the MADS-box protein, FcMADS9, induced anthocyanin biosynthesis, and ethylene promoted this effect [83]. 5-aminolevulinic acid (ALA) is a natural plant growth regulator that increases anthocyanin accumulation in apple skin. This ALA-induced anthocyanin biosynthesis required the function and activity of MdMADS1 [84].

### Indirect regulation through the MBW complex

Some TFs regulate the MBW complex activity by affecting transcription of *MYB*s. In Citrus, CitWRKY75 acted upstream of CitRuby1, and it promoted the transient accumulation of anthocyanin in various juvenile tissues [85]. A MADS-box TF, VmTDR4, was found to regulate MYBs in ripening bilberry and impact anthocyanin accumulation [86]. In the blood-flesh peach, NAC TFs, PpBL and PpNAC1, formed a heterodimer to activate *PpMYB10.1* gene, thus stimulating anthocyanin pigmentation [87]. The transactivation activity of the PpBL-PpNAC1 heterodimer was repressed by another TF, PpSPL1 [87].

Some TFs interact with MBW complex to regulate anthocyanin and PA biosynthesis. MdHB1 is an anthocyanin inhibitor in apple flesh [88]. MdHB1 could interact with MBW components to constrain the complex in the cytoplasm, thus repressing the transcription of *MdDFR* and *MdUFGT* [88]. VvMYC1 had the feedback regulation of its expression and interacted with various VvMYBs to induce anthocyanin and PA synthesis in the skin and seeds of grapevine berries [58].

**Figure 4:**
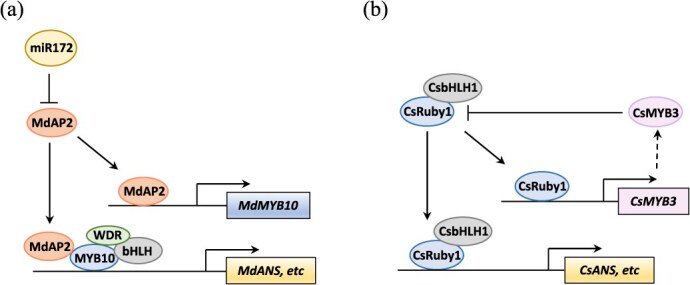
Indirect regulation of flavonoid biosynthesis through the MBW complex. (a) TF MdAP2 regulates the transcription of *MdMYB10* and interacts with the MBW complex component MYB10 to enhance anthocyanin biosynthesis. (b) Feedback regulation of the MBW complex by the induction of a repressor CsMYB3. Blunt ends represent inhibition effect.

As summarized in Fig. 4a, some TFs not only regulate the transcription of MBW components but also interact with them. MdAP2_1a positively regulated *MdMYB10* expression and it interacted with the MBW component MdMYB10 to promote anthocyanin biosynthesis in apples (Fig. 4a) [111]. Similarly, AcMADS68 regulated anthocyanin biosynthesis in kiwifruit flesh in two ways [89]. AcMADS68 interacted with AcMYBF110 and AcMYB123, stabilizing the formation of the MBW complex to upregulate anthocyanin-related genes. In addition, AcMADS68 directly activated the promoter of *AcbHLH1*, thus amplifying the MBW regulation signals and enhancing anthocyanin accumulation [89]. In red-skinned pears, PyWRKY26-PybHLH3 complex could co-target the *PyMYB114* promoter to promote the biosynthesis and transport of anthocyanin [90].

Interestingly, MBW complex has the feedback regulation to balance the anthocyanin in some fruit species. In citrus, CsMYB3 was transcriptionally activated by CsRuby1, and it repressed the transcriptional activity of the CsRuby1/CsbHLH1 complex [91]. Thus, CsRuby1 and CsMYB3 form an ‘activator-and-repressor’ loop to maintain the homeostasis of anthocyanin accumulation (Fig. 4b) [91]. Similarly, when grape berries were exposed to light, the VvHY5-VvMYBA1 module was activated, promoting anthocyanin biosynthesis [92]. After anthocyanin concentration reached a threshold level, VvMYBA1 activated the expression of *VvBBX44*, which in turn repressed the transcription of *VvHY5* and *VvMYBA1* [92]. In red-skinned pear fruits, PyMYB10/MYB114-PybHLH3 complex activated anthocyanin biosynthetic genes as well as *PyMYB107* [93]. PyMYB107 competitively bound PybHLH3 protein and interfered with MBW complex stability, thereby repressing excess anthocyanin production [93].

## Epigenetic regulation of flavonoid biosynthesis

**Figure 5:**
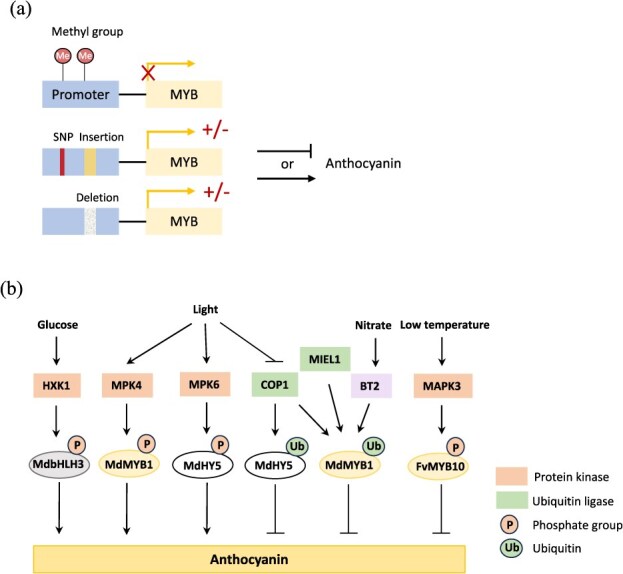
Epigenetic regulation and post-translational modification of TFs involved in the anthocyanin biosynthetic pathway. (a) Methylation of the promoter region of *MYB*s represses their transcription. SNPs, insertion, and deletion of DNA fragments in *MYB* promoters impact gene transcriptions, leading to either activation or inhibition of anthocyanin biosynthesis. SNPs, single nucleotide polymorphisms. (b) Regulation modes of protein phosphorylation and ubiquitination. Ubiquitin ligases promote the ubiquitination and degradation of anthocyanin activators, thereby inhibiting anthocyanin biosynthesis. Protein kinase-mediated phosphorylation modification of activators either enhances or attenuates their protein activity, thus impacting anthocyanin accumulation.

Epigenetic regulation is closely associated with the formation of diverse fruit color patterns. The underlying mechanisms involve the modification or variation of promoters of key genes regulating anthocyanin biosynthetic pathway (Fig. 5a). It was found that the *PpMYB10* promoter determined the striped pigmentation pattern of pear skin. The anthocyanin-rich tissues had lower methylation levels in the *PpMYB10* promoter region [61]. Similarly, the methylation level of the *MdMYB10* promoter was associated with apple skin pattern (blushed or striped). *MdMYB10* transcript levels in the red and green strips were inversely correlated with methylation levels in the promoter region [112]. Comparison of the anthocyanin-deficient yellow-skin apple mutant and its red-skin parent further revealed that the methylation levels of two regions in *MdMYB10* promoter negatively correlated with *MdMYB10*/*MdGST* expression and anthocyanin content [113]. The red-fleshed apple cultivar ‘Daihong’ had the color-fading phenomenon during fruit development. Decreased transcriptions of *MdMYBPA1*, *MdCHS*, *MdANS*, and *MdUFGT* were observed in the late development stage, consistent with the downregulation of chalcone and anthocyanin content [114]. Meanwhile, significant negative correlations were found between the promoter methylation levels of several flavonoid genes and their respective transcript levels, and the former also negatively correlated with downstream flavonoid contents [114]. The increase in DNA methylation was attributed to the decrease in expression of several DNA demethylase genes [114]. In figs (*Ficus carica*), a DNA methyltransferase FcMET1 was found that could mediate low DNA methylation of anthocyanin structural genes and promote peel coloring [115].

It is well known that transposon-induced epigenetic changes often alter proximal gene expressions. Multiple mechanisms have been revealed within these processes, including the disruption of promoter or coding sequences, the introduction of new alternative promoter sequences, and epigenetic silencing [116, 117]. The insertion of a long terminal repeat (LTR) retrotransposon upstream of *MdMYB1* regulated anthocyanin biosynthesis in the apple peel [118]. In the promoter of *MdWRKY10*, a functional 163-bp Indel was identified that controlled the degree of flesh red pigmentation in apples via transactivation of *MdWRKY10* and *MdMYB10* [94]. Mutations of *MYBA1* and *MYBA2* genes could cause the loss of grape skin pigmentation, leading to a ‘white’ phenotype. Insertion of a *Gret1* retrotransposon in *VvMYBA1* promoter suppressed its transcription, thereby suppressing anthocyanin accumulation in the berry skin [119]. In citrus, insertion of an LTR retrotransposon Tcs2 was found in the promoter of *Ruby*, an MYB transcriptional activator of anthocyanin. Tcs2 significantly increased *Ruby* transcript levels, and it conferred a red phenotype to the flesh of blood orange [120]. Interestingly, Tcs2 transcription was cold-inducible, which determined the cold dependency of *Ruby* expression and anthocyanin accumulation in the fruit [120]. Recently, it was found that *CsRuby1* was activated by two cold-responsive ethylene response factors (CsERF054 and CsERF061) via the retrotransposon in its promoter [121]. In a white-fruited strawberry ecotype, a gypsy transposon inserted in the coding region of *FaMYB10* truncated the protein and blocked anthocyanin biosynthesis. While a CACTA-like transposon inserted in the *FaMYB10-2* promoter elevated its expression and conferred strawberry a red flesh phenotype [122]. In peaches, a 487-bp deletion in the *PpMYB10.1* promoter enhanced its promoter activity. This deletion was found to be highly correlated with the red flesh phenotype [123]. Different pineapple varieties possess distinct red coloration patterns on the peels, and this is attributed to variation in the promoter regions of AcMYB266. Several single nucleotide polymorphisms (SNPs) and indels were discovered, and these differences determined the activation ability of these AcMYB266 promoters [124]. Moreover, AcMYB266 is located in a small gene cluster, in which four MYBs occur as two pairs of tandem genes. Each individual MYB regulated anthocyanin in specific pineapple tissues [124].

## Post-transcriptional regulation of flavonoid biosynthesis

Recent studies suggested the involvement of miRNAs and small interfering RNAs (siRNAs) in the regulation of fruit flavonoid biosynthesis [111, 125, 126]. MiRNAs are a class of noncoding small RNAs that negatively regulate gene expression, and they play critical roles in plant development and secondary metabolism. Targeting by miRNAs causes RNA degradation and the subsequent production of siRNAs. In grapes, miR828 and miR858 targeted repressor VvMYB114 to promote anthocyanin [125]. In stable apple and *Arabidopsis*, overexpression of miR172 reduced red coloration and anthocyanin content [112]. The target of miR172 in apple was MdAP2_1a (Fig. 4a) [112]. Recently, an apple dimple fruit viroid-derived siRNA (vsiR693) was identified, which negatively regulated anthocyanin biosynthesis by targeting mRNA of a bHLH TF, *MdPIF1*, for cleavage [126].

Alternative splicing transcription also plays a role in the accumulation of varied anthocyanin levels. CsTT8 modulated anthocyanin biosynthesis and its transport into vacuoles in blood orange. The alternative splicing transcript of CsTT8 (Δ15-CsTT8), with one exon skipped, negatively regulated pigmentation in the pulp or the peel. Δ15-CsTT8 could not interact with CsMYBs or be located in the nucleus, and it may have the negative feedback regulation of *CsTT8* expression, thus limiting anthocyanin accumulation [127].

## Post-translational regulation of flavonoid biosynthesis

Post-translational modification of TFs plays an important role in fine-tuning flavonoid accumulation in response to environmental signals. The mechanisms have been well demonstrated in apples in the aspect of anthocyanin regulation, and they include ubiquitination, phosphorylation, and sumoylation modification of key proteins (Fig. 5b). Ubiquitination is an enzymatic process that involves the covalent attachment of one or more ubiquitin monomers to the lysine residue of a substrate protein. Polyubiquitination is generally associated with protein degradation by the 26S proteasome system, and it plays a significant role in regulating protein homeostasis [128]. In apples, MdMYB1 accumulated in the light and was degraded in the dark. It regulated the accumulation and transport of anthocyanin into vacuoles [95, 129]. MdCOP1s interacted with and modulated the ubiquitination and degradation of MdMYB1. Therefore, MdCOP1s negatively regulated the peel coloration of apple fruits via the degradation of MdMYB1 [95]. MdMIEL1 is a ubiquitin E3 ligase that also negatively regulates anthocyanin accumulation by degrading MdMYB1 protein [130]. MdWRKY40 promoted wounding-induced anthocyanin biosynthesis by interacting with MdMYB1, and it underwent MdBT2-mediated degradation in the absence of wounding [96]. MdBT2 also promoted the ubiquitination and degradation of MdMYB1 to inhibit anthocyanin biosynthesis in response to nitrate [72]. Moreover, MdBT2 could negatively regulate anthocyanin and PA biosynthesis via the 26S proteasome-mediated degradation of MdMYB9 [131]. Generally, ubiquitination inhibits anthocyanin biosynthesis in apples by degradation of MYB activators.

Protein phosphorylation is the most common type of post-translational modification that involves adding phosphate groups to specific amino acid residues of proteins. Mediated by kinases, phosphorylation regulates the activity of one or more proteins in response to various extracellular stimuli (Fig. 5b). Light treatment induced two mitogen-activated protein kinases, MdMPK4 and MdMPK6, in apples [132]. Active MdMPK4 phosphorylated MdMYB1 to promote anthocyanin accumulation [132]. In parallel, MdMPK6 directly interacted with and activated MdHY5 via phosphorylation to increase the stability of MdHY5 and prevent it from MdCOP1-mediated degradation. Phospho-MdHY5 enhanced its binding to target anthocyanin-related genes [133]. Low temperature repressed anthocyanin accumulation in strawberry fruits, thus greatly reducing their coloration. FvMAPK3 was identified as an important regulator of this process, and it functions via two mechanisms. FvMAPK3 activity was induced by low temperature, and it phosphorylated FvMYB10 to reduce its transcriptional activity. FvMAPK3 also phosphorylated the rate-limiting enzyme CHS1 to enhance its proteasome-mediated degradation [134]. Exogenous glucose induced anthocyanin in apples via the hexokinase MdHXK1 and its phosphorylation target MdbHLH3. Phosphorylation modification stabilized MdbHLH3 protein and increased its transcriptional activity on anthocyanin biosynthetic genes, thereby promoting anthocyanin accumulation [135].

SUMOylation also regulates anthocyanin biosynthesis. Small ubiquitin-like modifier (SUMO) polypeptides can be attached to various intracellular target proteins to alter their function and/or location [136]. In red-skinned apples, the small ubiquitin-like modifier E3 ligase MdSLZ1 was found to directly sumoylate MdMYB1 protein under moderately low temperature conditions. Sumoylation of MdMYB1 increased its protein stability and upregulated anthocyanin biosynthesis under stress conditions [137].

## Regulation modules involved in flavonoid biosynthesis

Multiple regulation modules responding to environmental and hormonal cues have been identified for regulating flavonoids in fruits (Table 3). For instance, the regulation module UVR8-COP1-HY5-MYB cascade is responsible for ultraviolet-B light (UV-B)-induced anthocyanin and flavonol synthesis in apples [97]. In response to UV-B, UVR8 monomerized and interacted with COP1, which prevented COP1 from targeting HY5 for ubiquitination and protein degradation. HY5 interacted with MYB10 or MYB22 to activate the transcription of *FLS* and *CHS* [97]. The WRKY1-LNC409-ERF109 regulation cascade also regulates light-induced anthocyanin accumulation in apple fruit [82]. Light signal induced anthocyanin and PA accumulation via the MdHY5-mediated repression of *MdWRKY41*, which encoded a TF interacting with MdMYB16. The MdWRKY41-MdMYB16 complex repressed the transcription of downstream targets, including *MdMYB12*, *MdANR*, and *MdUFGT* [139]. MdMYB114 positively regulated anthocyanin biosynthesis and transport, and it was transactivated by MdbZIP4-like [143]. In grapevine, light-responsive TF VvHY5 bound to *VvMYB24* promoter to activate its transcription. Moreover, VvMYB24 interacted with VvMYBA1 to form a protein complex to upregulate the expression of anthocyanin structural genes [144]. In pears, it was demonstrated that ROS mediated high light-induced anthocyanin biosynthesis by the PuHB40-PuMYB123-like-PubHLH3 cascade [145]. Under high-light stress, ROS promoted TF *PuHB40* expression and maintained a high level of PuHB40 phosphorylation by suppressing the transcription of a protein phosphatase 2A (PP2A). The activated PuHB40 enhanced the transcription of *PuMYB123-like* and increased anthocyanin accumulation in pear seedlings [145].

**Table 3:** Regulation modules affecting flavonoid biosynthesis in fruits.

**Regulation module**	**Species**	**Regulatory effect**	**Reference**
UVR8-COP1-HY5-MYB	*M. domestica*	Anthocyanin (+), flavonol (+)	[97]
WRKY1-LNC499-ERF109	*M. domestica*	Anthocyanin (+)	[82]
PRT1/SMXL8-AGL9-HY5	*M. domestica*	Anthocyanin (+)	[138]
miR172-AP2-MYB10	*M. domestica*	Anthocyanin (−)	[111]
HY5-WRKY41-MYB	*M. domestica*	Anthocyanin (+), PA (+)	[139]
SCF^COI1^-JAZ-bHLH	*M. domestica*	Anthocyanin (+), PA (+)	[54]
SCF^TIR1^-Aux/IAA-ARF13	*M. domestica*	Anthocyanin (−)	[139]
SINA1/RGL2a-WRKY75/MYB308	*M. domestica*	Anthocyanin (−)	[140]
ABI5/bZIP44-bHLH3/MYB1	*M. domestica*	Anthocyanin (+)	[141]
EIL1-MYB1-ERF3	*M. domestica*	Anthocyanin (+)	[142]
bZIP4-like-MYB114	*M. domestica*	Anthocyanin (+)	[143]
HY5-MYB24-MYBA1	*V. vinifera*	Anthocyanin (+)	[144]
HB40-MYB123-like-bHLH3	*Pyrus ussuriensis*	Anthocyanin (+)	[145]
ERF054/061-Ruby1	*C. sinensis*	Anthocyanin (+)	[121]

The sign of plus (+) and minus (−) represents promotion and inhibition effects, respectively.

**Figure 6:**
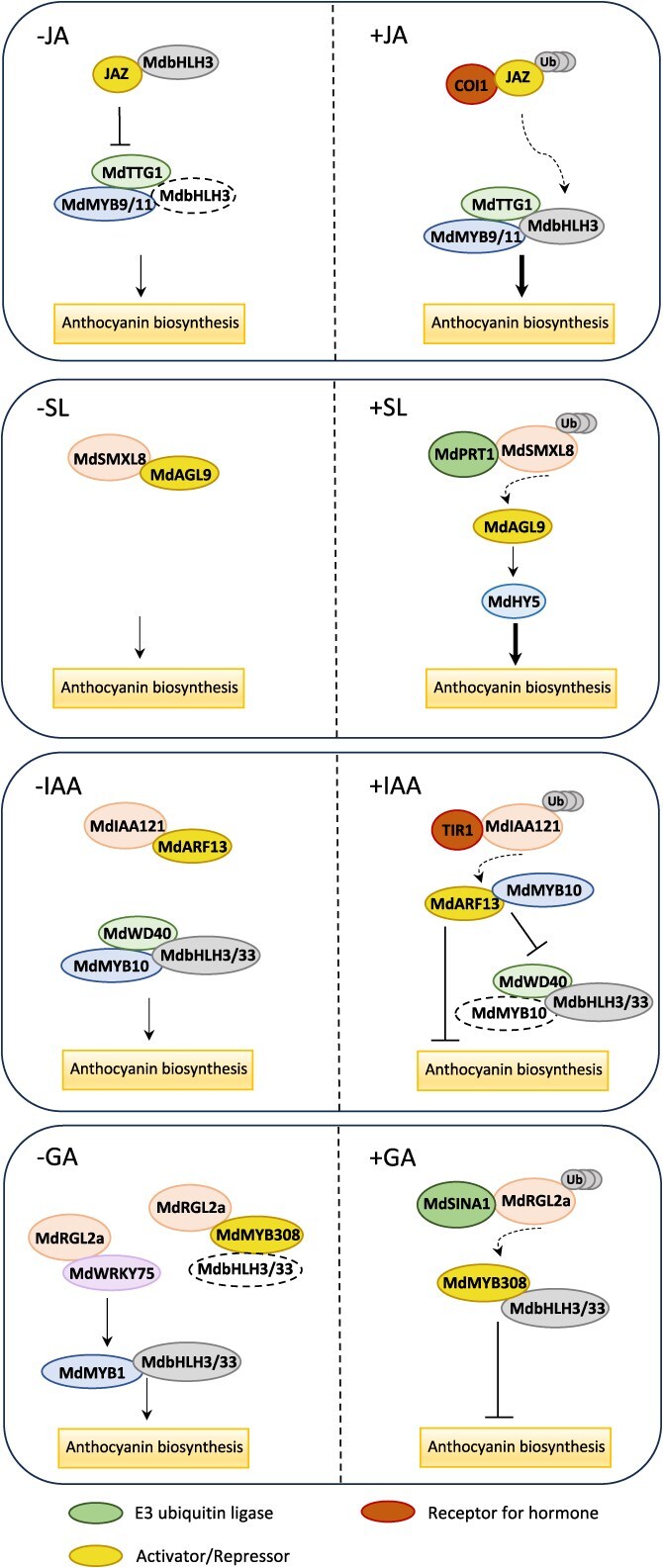
Hormone signal-induced cascade regulation of anthocyanin biosynthesis in fruits. Solid arrows and blunt ends represent the promotion and repression of anthocyanin biosynthesis, respectively. Line thickness of solid arrows indicates the activity of transcriptional activation. Dashed arrows indicate the release of either an activator or a repressor. JA, jasmonate; SL, strigolactone; IAA, indole-3-acetic acid; GA, gibberellic acid; Ub, ubiquitin.

Hormone signaling pathways significantly affect flavonoid biosynthesis in fruits (Fig. 6). Jasmonates (JAs) treatment increased anthocyanin and PA content in apples [56]. In the absence of JA signal, MdJAZ interacted with MdbHLH3 to attenuate the formation of an MBW complex. Upon perception of JA signal, MdJAZ was degraded, whereas MdbHLH3 was released to interact with MdMYBs and MdTTG1, forming the MBW complex to activate downstream structural genes [56]. The PRT1/SMXL8-AGL9-HY5 cascade promoted SL-mediated anthocyanin accumulation [138]. When the phytohormone strigolactones (SLs) were present, MdPRT1 mediated the ubiquitination and degradation of MdSMXL8, thus releasing MdAGL9 from the MdSMXL8-MdAGL9 inhibitory complex. Activated MdAGL9 promoted the following MdHY5-mediated anthocyanin biosynthesis [138]. Gibberellic acid (GA) signal negatively regulates anthocyanin biosynthesis in apples [140]. In the absence of GA, the DELLA protein MdRGL2a interacts with MdWRKY75 to enhance the MdWRKY75-activated transcription of *MdMYB1*. Meanwhile, MdRGL2a interacts with anthocyanin repressor MdMYB308 to release MdbHLH3/33. The MdMYB1-MdbHLH3/33 complex induces anthocyanin accumulation. Upon perception of GA signal, MdSINA1 mediates the ubiquitination and degradation of MdRGL2a, thus releasing MdMYB308 to inhibit anthocyanin biosynthesis [140]. Interestingly, MdRGL2a weakens the inhibitory effect of MdSMXL8 on anthocyanin biosynthesis by interfering with MdSMXL8–MdAGL9 interaction. Thus, the MdRGL2a and MdSMXL8 proteins mediate the crosstalk between GA and SL signaling [138]. Auxin signaling negatively regulates anthocyanin in apples by the Aux/IAA-ARF cascade [146]. Upon perception of exogenous auxin, the interaction within the MdIAA121-MdARF13 complex was disrupted by the degradation of MdIAA121, releasing MdARF13. As a negative regulator of the anthocyanin metabolic pathway, MdARF13 directly bound to the *MdDFR* promoter and inhibited MBW complex via interacting with MdMYB10 [146]. In climacteric fruits, the plant hormone ethylene initiates the ripening process. While in nonclimacteric fruits, such as strawberry, grapevine, and blueberry, ABA seems to regulate anthocyanin biosynthesis and ripening [147–149]. It was found that ethylene has the positive feedback regulation of anthocyanin biosynthesis in apples through an EIL1-MYB1-ERF3 module [142]. Through direct binding to the promoters, MdEIL1 transactivated *MdMYB1* to promote anthocyanin accumulation, and the latter transactivated *MdERF3* expression for enhancing ethylene production [142]. ABA also positively regulates anthocyanin biosynthesis in apples through the key regulators MdABI5 and MdbZIP44 [141]. ABA upregulated the expression of *MdbZIP44*, and MdABI5 transactivated *MdbHLH3*. The enhanced interactions within MdbZIP44-MdMYB1 and MdbHLH3-MdMYB1 complex collectively mediated ABA-induced anthocyanin accumulation [141].

## Metabolic engineering of flavonoid in fruits

Although fruits are rich in flavonoids, many of them contain only a few flavonoid classes (Table 1). Since their chemical structure, such as the basic skeleton and the modification patterns, determines the bioactivity of flavonoids, genetic engineering of fruits with modified flavonoid levels and composition has raised great interest. Tomato is the main model fruit used for metabolic engineering breeding. Transgenic tomatoes with higher levels of flavones accumulated in the fruit peel were produced, as well as those accumulating flavonol in the flesh by overexpressing maize regulatory genes *Lc* and *C1* [150, 151]. SlMYB75 regulates a set of tomato fruit quality traits [152]. The *SlMYB75*-OE fruits not only accumulated more anthocyanin, phenolics, and flavonoids but also displayed many physiological changes, such as increased ethylene production and delayed ripening [152]. Similarly, overexpression of *SlMX1*, an MYB TF, promoted many agroeconomic traits, such as fruit yield, quality, resistance to *Botrytis cinerea*, and biosynthesis of anthocyanin and other flavonoids [153]. Lately, increased production of anthocyanin in apple plants and pineapple peel was achieved by overexpressing *MdMYB10* and *AcMYB266*, respectively [124, 154].

Flavonoid regulatory and structural genes from other horticultural plants were also utilized for tomato breeding through synthetic biology. *CnFLS1* was identified as the key structural gene in the biosynthetic pathway of quercetin 3-*O*-glucoside and quercetin 7*-O*-glucoside in Golden *Camellia* [155]. These quercetin derivatives confer *Camellia* golden pigmentation in flowers. Transformation of *CnFLS1* and other flavonol structural genes yielded yellow tomato fruit with quercetin-enriched flesh [155]. These distinct flavonol components derived from golden *Camellia* not only alter the appearance of tomato fruit but also significantly increase its antioxidant capabilities and health benefits [155].

Advances of flavonoid regulation in some fruits provide future targets for molecular breeding. For instance, SlMYB7 is a negative regulator of anthocyanin in ‘black pearl’ tomato fruits, which acts by repressing *SlANS* expression and interacting with bHLH proteins, thus providing a new molecular breeding target [156]. Other flavonoid regulatory factors in tomatoes include phosphate deficiency-induced SlPHL1 that positively regulates anthocyanin [157], and SlMYB72 that negatively regulates flavonol accumulation in pericarps [158]. The WD40 gene, *AcTTG1*, and the R2R3 MYB *AcMYBF110* could be candidate loci in kiwifruit as they promote anthocyanin biosynthesis in tobacco leaves [159]. It’s well known that nitrogen or phosphorus fertilization supplies nutrients for plants, and it greatly influences fruit quality, especially anthocyanin formation [160]. Therefore, the network of nutrient signaling has the potential to positively regulate anthocyanin levels, and key components therein can be engineered in important commercial fruits, such as grapes, apples, and strawberries.

In recent years, CRISPR/Cas9 editing system has been used in tomatoes and grapes for probing potential flavonoid regulatory genes [161]. In grapes, stilbene synthase (STS) and CHS catalyze the same substrates and compete to produce resveratrol and flavonoids, respectively. The knockout of *CHS2* gene in *Vitis davidii* cells downregulated flavonoid accumulation and shifted the metabolic flow toward the biosynthesis of resveratrol [162]. Yet, the use of this genome editing technology has rarely been reported on other fruits for engineering flavonoids [163]. This is partly due to the difficulty of developing available transformation approaches. Moreover, the heterozygosity and polyploidy of the fruit genome, as well as the long juvenile stage, pose challenges to experiment and optimize genome editing. The low editing efficiency of the traditional CRISPR/Cas9 system and the emergence of somatic mosaics make it difficult to identify heritable mutations. Meanwhile, robust nontransgenic genome editing methods need to be developed for fruit crops to reduce public resistance to genetically modified organisms. Currently, the scientific community has developed some methods to address these challenges. For example, the use of the polycistronic tRNA-gRNA (PTG)/Cas9 system ensures the simultaneous production of multiple gRNAs targeting different genomic sites. This method uses the endogenous tRNA processing system to cut and release individual gRNAs from tandem tRNA-gRNA coding units [164]. The PTG/Cas9 system was utilized to edit the *PDS* (Phytoene Desaturase) gene in kiwifruit, achieving an efficiency 10 times higher than that of the traditional CRISPR/Cas9 [165]. Another multigene editing system, pYLCRISPR/Cas9, can simultaneously target five key genes of the γ-aminobutyric acid (GABA) shunt in tomato, creating single to quadruple mutants with significantly increased GABA content [166]. Nontransgenic genome editing has been pioneered in grapes [167]. One of these methods can directly introduce purified CRISPR/Cas9 ribonucleoproteins (RNPs) into protoplasts. This genome editing process does not involve any foreign DNA, as the RNPs are subsequently degraded [167]. Short heat stress has been reported to increase editing efficiency, which may be related to the increase of gRNA expression level and/or the activity of some Cas9 enzymes [168].

## Conclusions and future perspective

Flavonoids have significant bioactivity and are widely present in horticultural crops. Research regarding the synthesis and regulation of flavonoids has increased significantly, which involves complex regulatory networks such as direct or indirect regulation by different types of TFs, miRNA-mediated post-transcriptional regulation, epigenetic regulation, post-translational modifications, and transcriptional regulatory cascades. These studies have revealed the molecular mechanisms of the regulation of flavonoid synthesis under specific environmental conditions or hormonal signals. However, research gaps remain in many other aspects. For instance, research addressing the cross-talks of different exogenous and endogenous signals is lacking. Despite the general conservation of the MBW complex’s regulatory mechanisms, notable differences in their sensitivity to specific hormonal responses and variations may exist, leading to varied downstream products between apples and grapes and reflecting their adaptive strategies and evolutionary histories. Although anthocyanin regulation was extensively studied, research on other bioactive flavonoids, such as flavonol, flavone, and flavanones, is still limited. Flavonoids, in terms of types, functions, and regulatory mechanisms, are largely unknown in many medical and unconventional fruits. Structural modifications of flavonoids, such as hydroxylation and glycosylation, can alter the flavor and bioactivity of the substance. For instance, novel 7-*O*-glucosyltransferase genes (7GlcTs) identified in citrus were capable of glycosylation at the 7-hydroxy position of various flavonoids [169]. These 7GlcTs promoted the accumulation of flavonoid glycosides and could play a role in the defense of citrus against Huanglongbing infection [169]. Research on such genes involved in structural modifications requires further exploration.

Condition-specific transcriptomic and metabolomic analysis in different crop species will advance the probe of the regulatory gene network associated with flavonoid synthesis. Further functional studies, which involve transgenesis, mutant analysis, and molecular interactions, should verify the key molecular players involved in regulating flavonoid biosynthesis of new horticultural crops. Single-cell sequencing technology can be utilized to study the heterogeneous regulation of complex biochemical pathways at the cellular level. It can help to elucidate the specific metabolic pathways of flavonoid compounds in different cell types, organs, or developmental stages. Combined with spatial transcriptomics, single-cell sequencing technology can achieve precise localization of flavonoid synthesis in specific regions of the fruits. For instance, the spatiotemporal trajectories of senescence were revealed within the pericarp of pitaya by single-cell and spatial RNA sequencing [170]. Early-stage oxidative stress occurred initially in the mesocarp. This was followed by a significant activation of resistance genes in the exocarp cells, including those involved in flavonoid biosynthesis. This study reinforces the belief that flavonoids could serve as a marker for fruit senescence [170]. Single-cell and spatial transcriptomics technologies can be applied to demonstrate a series of biological processes, including the mechanisms involved in crop transformation, organ development, disease resistance, abiotic stress, and yield [171]. Yet these aspects were elucidated mainly in crops such as maize, wheat, rice, and cotton, and it remains to be explored in fruits [171].

Increasing the flavonoid content in fruits through genetic selection, bioengineering, or physical treatments not only benefits human health but may also improve fruit quality by manipulating color and flavor. Enhanced appearance quality of economically valuable fruits can help to increase their added value and market competitiveness. In addition, molecular breeding for improved synthesis of flavonoid can reduce pesticide use and lower environmental risks, especially in fruit crops such as apples and strawberries. The antioxidant capacity and nutritional value of engineered fruits can be significantly increased, thereby providing new functional food for the consumption of flavonoids. This review provides valuable insights for using molecular design breeding and synthetic biology to increase flavonoid production and/or improve the appearance and flavor quality of horticultural crops, thereby facilitating flavonoid consumption for public health benefit.

## Data Availability

The authors confirm that all data in this study are available and can be found in this article.
